# Impact of foliar application of syringic acid on tomato (*Solanum lycopersicum* L.) under heavy metal stress-insights into nutrient uptake, redox homeostasis, oxidative stress, and antioxidant defense

**DOI:** 10.3389/fpls.2022.950120

**Published:** 2022-08-25

**Authors:** Jing Ma, Muhammad Hamzah Saleem, Baber Ali, Rizwan Rasheed, Muhammad Arslan Ashraf, Humera Aziz, Sezai Ercisli, Sana Riaz, Mohsen Mohamed Elsharkawy, Iqbal Hussain, Sadeq K. Alhag, Ahmed Ezzat Ahmed, Dan C. Vodnar, Sahar Mumtaz, Romina Alina Marc

**Affiliations:** ^1^School of Public Administration, Hohai University, Nanjing, China; ^2^College of Plant Science and Technology, Huazhong Agricultural University, Wuhan, China; ^3^Department of Plant Sciences, Quaid-i-Azam University, Islamabad, Pakistan; ^4^Department of Botany, Government College University, Faisalabad, Pakistan; ^5^Department of Environmental Sciences and Engineering, Government College University, Faisalabad, Pakistan; ^6^Department of Horticulture, Faculty of Agriculture, Ataturk University, Erzurum, Turkey; ^7^Department of Bioinformatics and Biotechnology, Government College University, Faisalabad, Pakistan; ^8^Department of Agricultural Botany, Faculty of Agriculture, Kafrelsheikh University, Kafr El-Sheikh, Egypt; ^9^Department of Biology, College of Science and Arts, King Khalid University, Muhayl Asser, Saudi Arabia; ^10^Department of Biology, College of Science, Ibb University, Ibb, Yemen; ^11^Department of Biology, College of Science, King Khalid University, Abha, Saudi Arabia; ^12^Department of Theriogenology, Faculty of Veterinary Medicine, South Valley University, Qena, Egypt; ^13^Institute of Life Sciences, Faculty of Food Science and Technology, University of Agricultural Sciences and Veterinary Medicine, Cluj-Napoca, Romania; ^14^Division of Science and Technology, Department of Botany, University of Education, Lahore, Pakistan; ^15^Food Engineering Department, Faculty of Food Science and Technology, University of Agricultural Science and Veterinary Medicine Cluj-Napoca, Cluj-Napoca, Romania

**Keywords:** contamination, ROS, antioxidants, oxidative stress, foliar application

## Abstract

Soil contamination with toxic heavy metals [such as lead (Pb)] is becoming a serious global problem due to the rapid development of the social economy. However, accumulation of Pb in plant parts is very toxic for plant growth and decreases crop yield and productivity. In the present study, we have investigated the different concentrations of Pb in the soil i.e., [0 (no Pb), 50, and 100 mg kg^–1^] to study plant growth and biomass, photosynthetic pigments and gas exchange characteristics, oxidative stress indicators and the response of various antioxidants (enzymatic and non-enzymatic), nutritional status of the plant, organic acid exudation pattern and also Pb accumulation in the roots and shoots of the plants of two varieties of tomato (*Solanum lycopersicum* L.) i.e., Roma and Cchuas, grown under different levels of synergic acid [no spray (NS), water spray (WS), 0.3-0.5°μM]. Results from the present study showed that the increasing levels of Pb in the soil decreased non-significantly (*P* < 0.05) shoot length, root length, shoot fresh weight, root fresh weight, shoot dry weight, root dry weight, chlorophyll-a, chlorophyll-b, total chlorophyll, carotenoid content, net photosynthesis, stomatal conductance, transpiration rate, soluble sugar, reducing sugar, non-reducing sugar contents, calcium (Ca^2+^), magnesium (Mg^2+^), iron (Fe^2+^), and phosphorus (P) contents in the roots and shoots of the plants. However, Pb toxicity also induced oxidative stress in the roots and shoots of the plants by increasing malondialdehyde (MDA), hydrogen peroxide (H_2_O_2_), and electrolyte leakage (EL) which also induced increased the compounds of various enzymatic and non-enzymatic antioxidants and also organic acids exudation pattern in the roots such as fumaric acid, acetic acid, citric acid, formic acid, malic acid, oxalic acid contents and increased the concentration of Pb in different parts of the plants. Results also show that the Cchuas showed better growth and development compared to Roma, under the same levels of Pb in the soil. The alleviation of Pb toxicity was induced by the application of synergic acid, and results showed that the application of synergic acid increased plant growth and biomass and also increased the gas exchange characteristics and antioxidant capacity in the roots and shoots of the plants. Research findings, therefore, suggested that synergic acid application can ameliorate Pb toxicity in *S. lycopersicum* varieties and result in improved plant growth and composition under metal stress as depicted by balanced exudation of organic acids.

## Introduction

Heavy metals are omnipresent pollutants of the environment, and their excessive level in arable soil can pose serious threats not only to normal plant growth and development but also to human health (Nawaz et al., 2017, 2022; Alamri et al., 2018; Deng et al., 2021). The release of toxic heavy metals in agricultural soil due to anthropogenic activities (mining, wastewater sludge, and pesticides) is the major cause of global heavy metal pollution (Gill et al., 2013; Saleem et al., 2020a; Irshad et al., 2021). Heavy metals are significant environmental pollutants, and their toxicity is a problem of increasing significance for ecological, evolutionary, nutritional, and environmental reasons (Nagajyoti et al., 2010; Riaz et al., 2020; Zainab et al., 2021). Among different heavy metals, lead (Pb) contamination in the soil lasts for 150–5,000 years and is hard to remediate, resulting in long-term accumulation in soil and organisms (Uddin et al., 2016; Deng et al., 2021). Moreover, Pb toxicity in plants is associated with negative effects on nutrient uptake, photosynthesis, and antioxidant enzymes (Shahid et al., 2014; Tang et al., 2020). Generally, it is very difficult to decontaminate Pb-polluted fields by using plants alone, because metal is frequently accumulated in surface soil layers and only a small portion is present in soil solutions (Hadi et al., 2010; Kanwal et al., 2014). Antioxidant enzymes are one mechanism plants have evolved as a response to metal-induced toxicity (Ahmad et al., 2022b; Naveed et al., 2022; Wahab et al., 2022). For example, guaiacol peroxidase (POD), ascorbate peroxidase (APX), superoxide dismutase (SOD), and catalase (CAT) significantly contribute to regulating the cellular redox homeostasis to a safe level (Saleem et al., 2020c; Afzal et al., 2021; Amna et al., 2021; Yasmin et al., 2021; Khan et al., 2022). However, under Pb toxicity, overproduction of reactive oxygen species (ROS) disturbs this redox equilibrium (Farid et al., 2013; Mallhi et al., 2019; Mobeen et al., 2021). ROS-induced lipid membrane peroxidation and oxidative stress interrupt normal metabolic activities and damage biological molecules such as proteins, lipids, and nucleic acids; ultimately leading to cellular destruction (Rehman et al., 2019; Saleem et al., 2020b; Chen et al., 2021; Xiong et al., 2021). Clearly, a viable and cost-effective method to remove Pb from the environment is needed.

Syringic acid (SA) is a phenolic compound often found in fruits and vegetables and which is synthesized *via* the shikimic acid pathway in plants (Vo et al., 2020). Syringic acid is distributed in a wide variety of plant products (fruits and vegetables) and certain fungal species and also possesses natural derivatives, which occur along with Syringic acid in plants (Srinivasulu et al., 2018). It is an off-white powder and soluble in ethanol or methanol or ethyl ether; however, it is also slightly soluble in water [5780 mg/L (25°C)]. The other names of Syringic acid are 4-Hydroxy-3, 5-dimethoxybenzoic acid; 3, 5-Dimethoxy-4-hydroxybenzoic acid; Cedar acid; Gallic acid 3, 5-dimethyl ether with the chemical formula is C_9_H_10_O_5_ (Srinivasulu et al., 2018). Previously, it was investigated that cesium toxicity is alleviated by the exogenous application of syringic acid in *Arabidopsis* (Adams et al., 2020). Tomato (*Solanum lycopersicum* L.), is widely cultivated in tropics, subtropics, and warm temperate regions and has been recognized as an essential vegetable crop consumed by human beings across the globe (Ali et al., 2020; Dad et al., 2021). It is a rich source of lycopene, phenolics, and flavonoids that act as chemo-protective compounds and play first-line defense against various chronic diseases. *S. lycopersicum* seed contains melatonin around 5 to 114,500°pg g^–1^ on a fresh weight basis (Kamran et al., 2020). In Pakistan, tomato is cultivated on over 54,000 hectares, comprising over 25% of the total area under vegetable cultivation (Ali et al., 2020). The *S. lycopersicum* fields are at higher risk of harsh environmental stresses such as flooding, salinity, drought, and heavy metals including Pb. Plants with high biomass production and greater tolerance, such as *S. lycopersicum* are highly capable of extracting large quantities of Pb by depositing sufficient amounts of Pb concentrations in their roots and shoots. As far as the authors’ understand, very few studies have investigated the Pb-induced morpho-physiological and biochemical changes in *S. lycopersicum* plants and its amelioration through various growth modulators or phytochemicals. Therefore, the current study was carried out to evaluate how Syringic acid induces tolerance in *S. lycopersicum* varieties against severe Pb toxicity. It was hypothesized that the application of Syringic acid may alleviate the Pb-induced oxidative damage by reducing the Pb uptake in *S. lycopersicum* varieties (Roma and Cchuas). The main objectives of this study were (i) to determine the tolerance induction efficiency and application rate of Syringic acid when applied to Pb-stressed *S. lycopersicum* varieties; (ii) to measure the role of Syringic acid accelerated variations on morphological, physiological, and biochemical levels and antioxidant enzyme activities in *S. lycopersicum* varieties against Pb toxicity. Moreover, these findings will elaborate new insights into understanding the mechanisms responsible for enhanced Pb tolerance in *S. lycopersicum* varieties under Syringic acid phytochemicals. The results from the present study gave a new insight that the use of Syringic acid in heavy metals studies may be beneficial and can improve plant yield under Pb-contaminated soil.

## Materials and methods

### Plant material and cultivation

The present study was conducted in the botanical garden under a greenhouse environment belonging to the Department of Botany, Government College University, Faisalabad 38000, Punjab, Pakistan (31° 24/N, 73° 04/E). Healthy and mature seeds of two Tomato (*Solanum lycopersicum* L.) varieties (Roma and Cchuas) were collected from Ayub Agricultural Research Institute (AARI) Faisalabad, Punjab, Pakistan. Previously, *S. lycopersicum* has been grown as a potential vegetative plant under Pb polluted soil (Irfan et al., 2010; Pérez et al., 2013). The soil used for this experiment was collected from the experimental station of Government College University, Faisalabad, and was air dried, passed through a 5-mm sieve, and was water saturated twice before being used in pots. The physio-chemical properties of the soil used in this study are presented in Supplementary Table 1. Before sowing, the seeds were surface sterilized with 0.1% HgCl_2_ for the prevention of any surface fungal/bacterial contamination (Mohamed et al., 2020).

### Experimental layout

Small pots (15 cm height × 20 cm width) were used in this study, each containing 5 kg of uncontaminated soil. Before sowing seeds in the pots, the soil was spiked artificially with Pb using lead chloride (PbCl_2_) at various levels i.e., [0 (no Pb), 50, and 100 mg kg^–1^] and equilibrated for 60°days by four cycles of saturation with d.H_2_O and air drying. After seed germination (more than 2 cm emergence) (Saleem et al., 2019), the plants were exogenously supplied (once in the whole experiment) with various levels of spray: (1) no spray, (2) water spray, (3) syringic acid (0.3°μM), and (4) syringic acid (0.5°μM). Two varieties of *S. lycopersicum* have been used in this experiment and were designed in such a way that total 12 treatments were used in this study; (1) 0 mg kg^–1^ Pb concentration + no spray, (2) 0 mg kg^–1^ Pb concentration + water spray, (3) 0 mg kg^–1^ Pb concentration + syringic acid (0.3°μM), (4) 0 mg kg^–1^ Pb concentration + syringic acid (0.5°μM), (5) 50 mg kg^–1^ Pb concentration + no spray, (6) 50 mg kg^–1^ Pb concentration + water spray, (7) 50 mg kg^–1^ Pb concentration + syringic acid (0.3°μM), (8) 50 mg kg^–1^ Pb concentration + syringic acid (0.5°μM), (9) 100 mg kg^–1^ Pb concentration + no spray, (10) 100 mg kg^–1^ Pb concentration + water spray, (11) 100 mg kg^–1^ Pb concentration + syringic acid (0.3°μM), and (12) 100 mg kg^–1^ Pb concentration + syringic acid (0.5°μM). The level of Pb concentration used in this experiment was less than in the studies of Zafar-ul-Hye et al. (2020), Deng et al. (2021). All pots were placed in a completely randomized design (CRD) having five plants in each pot with four replicates of each treatment. The total duration of experimental treatments was 6°weeks under controlled conditions. Irrigation with Pb-free water and other intercultural operations were performed when needed. All plants in the glass house territory received natural light, with a day/night temperature of 35/40°C and day/night humidity of 60/70%. Although this experiment was conducted in pots, for the collection of organic acids, two seedlings were transferred to rhizoboxes which consisted of plastic sheet, nylon net, and wet soil (Javed et al., 2013). After 48 h, plants were taken from the rhizoboxes and the roots were washed with redistilled water to collect the exudates from the root surface. The samples were filtered through a 0.45°μm filter (MillexHA, Millipore, United States) and collected in Eppendorf tubes (Greger and Landberg, 2008). The collected samples were mixed with NaOH (0.01 M) in order to analyze the organic acids. However, the samples used for the analysis of oxalic acid were not treated with NaOH (Javed et al., 2013).

### Plant harvesting

After 6°weeks, the remaining three seedlings were up-rooted and washed gently with the help of distilled water to eliminate the aerial dust and deposition. The functional leaf in each treatment was picked at a rapid growth stage during 09:00–10:30 AM. The sampled leaves were washed with distilled water, immediately placed in liquid nitrogen, and stored in a freezer at –80°C for further analysis. All the harvested plants were divided into two parts i.e., roots and shoots to study different physio-biochemical traits. Leaves from each treatment group were picked for chlorophyll, carotenoid, oxidative stress, and antioxidants analysis. Root and shoot lengths were measured straightway after the harvesting by using a measuring scale and digital weighing balance to measure fresh biomass. Roots were uprooted and immersed in 20 mM Na_2_EDTA for 15–20 min to remove Pb adhered to the root surfaces. Then, roots were washed thrice with distilled water and finally once with de-ionized water and dried for further analysis. The different parts of the plant (roots and shoots) were oven-dehydrated at 65°C for 72 h for Pb determination and the total plant dry weight was also measured.

### Determination of photosynthetic pigments and gas exchange characteristics

Leaves were collected for the determination of chlorophyll and carotenoid contents. For chlorophylls, 0.1 g of fresh leaf sample was extracted with 8 mL of 95% acetone for 24 h at 4°C in the dark. The absorbance was measured by a spectrophotometer (UV-2550; Shimadzu, Kyoto, Japan) at 646.6, 663.6, and 450 nm. Chlorophyll content was calculated by the standard method of Arnon (1949).

Net photosynthesis (*Pn*), leaf stomatal conductance (*Gs*), transpiration rate (*Ts*), and intercellular carbon dioxide concentration (*Ci*) were measured from four different plants in each treatment group. Measurements were conducted between 11:30 and 13:30 on days with a clear sky. Rates of leaf *Pn*, *Gs, Ts*, and *Ci* were measured with an LI-COR gas-exchange system (LI-6400; LI-COR Biosciences, Lincoln, NE, United States) with a red-blue LED light source on the leaf chamber. In the LI-COR cuvette, CO_2_ concentration was set as 380 mmol mol^–1^, and LED light intensity was set at 1,000 mmol m^–2^ s^–1^, which was the average saturation intensity for photosynthesis in *S. lycopersicum* (Austin, 1990).

### Determination of oxidative stress indicators

The degree of lipid peroxidation was evaluated as malondialdehyde (MDA) contents. Briefly, 0.1 g of frozen leaves were ground at 4°C in a mortar with 25 mL of 50 mM phosphate buffer solution (pH 7.8) containing 1% polyethylene pyrrole. The homogenate was centrifuged at 10,000 × *g* at 4°C for 15 min. The mixtures were heated at 100°C for 15–30 min and then quickly cooled in an ice bath. The absorbance of the supernatant was recorded by using a spectrophotometer (xMark™ Microplate Absorbance Spectrophotometer; Bio-Rad, United States) at wavelengths 532, 600, and 450 nm. Lipid peroxidation was expressed as l mol g^–1^ by using the formula: 6.45 (A532–A600)−0.56 A450. Lipid peroxidation was measured by using a method previously published by Heath and Packer (1968) and Mehmood et al. (2021).

To estimate H_2_O_2_ content of plant tissues (root and leaf), 3 mL of sample extract was mixed with 1 mL of 0.1% titanium sulfate in 20% (v/v) H_2_SO_4_ and centrifuged at 6,000 × *g* for 15 min. The yellow color intensity was evaluated at 410 nm. The H_2_O_2_ level was computed by the extinction coefficient of 0.28 mmol^–1^ cm^–1^. The contents of H_2_O_2_ were measured by the method presented by Jana and Choudhuri (1981) and Ali et al. (2022a).

Stress-induced electrolyte leakage (EL) of the uppermost stretched leaves was determined by using the methodology of Dionisio-Sese and Tobita (1998) and Ma et al. (2022). The leaves were cut into minor slices (5 mm in length) and placed in test tubes containing 8 mL of distilled water. These tubes were incubated and transferred into a water bath for 2 h prior to measuring the initial electrical conductivity (EC_1_). The samples were autoclaved at 121°C for 20 min and then cooled down to 25°C before measuring the final electrical conductivity (EC_2_). Electrolyte leakage was calculated by the following formula;


$$\mathrm{EL}=\left({\mathrm{EC}}_{1}/{\mathrm{EC}}_{2}\right)\times 100$$

### Determination of antioxidant enzyme activities

To evaluate enzyme activities, fresh leaves (0.5 g) were homogenized in liquid nitrogen and 5 mL of 50 mmol sodium phosphate buffer (pH 7.0), including 0.5 mmol EDTA and 0.15 mol NaCl. The homogenate was centrifuged at 12,000 × *g* for 10 min at 4°C, and the supernatant was used for measurement of superoxidase dismutase (SOD) and peroxidase (POD) activities. SOD activity was assayed in a 3 mL reaction mixture containing 50 mM sodium phosphate buffer (pH 7), 56 mM nitro blue tetrazolium, 1.17 mM riboflavin, 10 mM methionine, and 100°μL enzyme extract. Finally, the sample was measured by using a spectrophotometer (xMark™ Microplate Absorbance Spectrophotometer; Bio-Rad). Enzyme activity was measured by using a method by Chen and Pan (1996) and expressed as U g^–1^ FW.

Peroxidase (POD) activity in the leaves was estimated by using the method of Sakharov and Ardila (1999) by using guaiacol as the substrate. A reaction mixture (3 mL) containing 0.05 mL of enzyme extract, 2.75 mL of 50 mM phosphate buffer (pH 7.0), 0.1 mL of 1% H_2_O_2_, and 0.1 mL of 4% guaiacol solution was prepared. Increases in the absorbance at 470 nm because of guaiacol oxidation was recorded for 2 min. One unit of enzyme activity was defined as the amount of the enzyme.

Catalase (CAT) activity was analyzed according to Aebi (1984). The assay mixture (3.0 mL) was comprised of 100°μL enzyme extract, 100°μL H_2_O_2_ (300 mM), and 2.8 mL 50 mM phosphate buffer with 2 mM ETDA (pH 7.0). The CAT activity was measured from the decline in absorbance at 240 nm as a result of H_2_O_2_ loss (ε = 39.4 mM^–1^ cm^–1^).

Ascorbate peroxidase (APX) activity was measured according to Nakano and Asada (1981) and Ali et al. (2022c). The mixture containing 100°μL enzyme extract, 100°μL ascorbate (7.5 mM), 100°μL H_2_O_2_ (300 mM), and 2.7 mL 25 mM potassium phosphate buffer with 2 mM EDTA (pH 7.0) was used for measuring APX activity. The oxidation pattern of ascorbate was estimated from the variations in wavelength at 290 nm (ε = 2.8 mM^–1^ cm^–1^).

### Determination of non-enzymatic antioxidants, sugars, and proline contents

Plant ethanol extracts were prepared for the determination of non-enzymatic antioxidants and some key osmolytes. For this purpose, 50 mg of dry plant material was homogenized with 10 mL ethanol (80%) and filtered through Whatman No. 41 filter paper. The residue was re-extracted with ethanol, and the two extracts were pooled together to a final volume of 20 mL. The determination of flavonoids (Pękal and Pyrzynska, 2014), phenolics (Bray and Thorpe, 1954), ascorbic acid (Azuma et al., 1999), anthocyanin (Lewis et al., 1998), and total sugars (Dubois et al., 1956) and also free amino acids was performed from the extracts.

Fresh leaf material (0.1°g) was mixed thoroughly in 5 mL of aqueous sulphosalicylic acid (3%). The mixture was centrifuged at 10,000 × *g* for 15 min, and an aliquot (1 mL) was poured into a test tube having 1 mL acidic ninhydrin and 1 mL glacial acetic acid. The reaction mixture was first heated at 100°C for 10 min and then cooled in an ice bath. The reaction mixture was extracted with 4 mL toluene, and test tubes were vortexed for 20 s and cooled. Thereafter, the light absorbance at 520 nm was measured by using a UV–VIS spectrophotometer (Hitachi U-2910, Tokyo, Japan). The free proline content was determined on the basis of the standard curve at 520 nm absorbance and expressed as μmol (g FW) ^–1^ (Bates et al., 1973; Ali et al., 2022b).

### Determination of nutrient content

For nutrient analysis, plant roots and shoots were washed twice in redistilled water, dipped in 20 mM EDTA for 3 s, and then, again, washed with deionized water twice for the removal of adsorbed metal on the plant surface. The washed samples were then oven-dried for 24 h at 105°C. The dried roots and shoots were digested by using a wet digestion method in HNO_3_: HClO_4_ (7:3 V/V) until clear samples were obtained. Each sample was filtered and diluted with redistilled water up to 50 mL. The root and shoot contents of Fe, Ca, Mg, and P were analyzed by using Atomic Absorption Spectrophotometer (AAS) model Agilent 240FS-AA.

### Determination of root exudates analysis and Pb concentration

In order to determine the concentration of organic acids, freeze-dried exudates were mixed with ethanol (80%), and 20°μL of the solutions were injected into the C18 column (Brownlee Analytical C-183 μm; length 150 mm × 4.6°mm^2^, United States). Quantitative analysis of organic acids in root exudates was executed with high-performance liquid chromatography (HPLC), having a Flexer FX-10 UHPLC isocratic pump (PerkinElmer, MA, United States). The mobile phase used in HPLC was comprised of an acidic solution of aceto-nitrile containing aceto-nitrile:H_2_SO_4_:acetic acid in ratios of 15:4:1, respectively, and pH of 4.9. The samples were analyzed at a flow rate of 1.0 mL min^–1^ for a time period of 10 min. The inner temperature of the column was fixed at 45°C, and quantification of organic acids was carried out at 214 nm wavelength with the help of a detector (UV–VIS Series 200, United States) as described by UdDin et al. (2015). Freeze-dried samples were dissolved in redistilled water, and the pH of the exudates was recorded with LL micro-pH glass electrode by using a pH meter (ISTEK Model 4005–08007, Seoul, South Korea).

After 6°weeks of treatment, plants were harvested, cleansed with tap water, distilled water, and deionized water comprehensively. Immediately after that, the plant samples were differentiated into roots and shoots, dehydrated at 80°C in an oven for 48 h, and then mashed into powder. 0.5 g of each sample was dry-ash, extracted with HCl, and then centrifuged at 3,600 rpm for 15 min. Concentrations of Pb in roots and shoots were examined by flame atomic absorption spectrometry.

### Statistical analysis

The normality of data was analyzed using IBM SPSS software (Version 21.0, Armonk, NY, United States: IBM Corp) through a Tukey’s test to determine the interaction among significant values. Thus, the differences between treatments were determined by using ANOVA, and the least significant difference test (*P* < 0.05) was used for multiple comparisons between treatment means where significant. The graphical presentation was carried out using Origin-Pro 2019. The Pearson correlation coefficients between the measured variables of *S. lycopersicum* were also calculated. The plots of principal component analysis on *S. lycopersicum* parameters were carried out using the RStudio software.

## Results

### Effect of exogenous application of syringic acid on plant growth and photosynthetic measurements in *Solanum lycopersicum* varieties under the toxic concentrations of Pb

**FIGURE 1:**
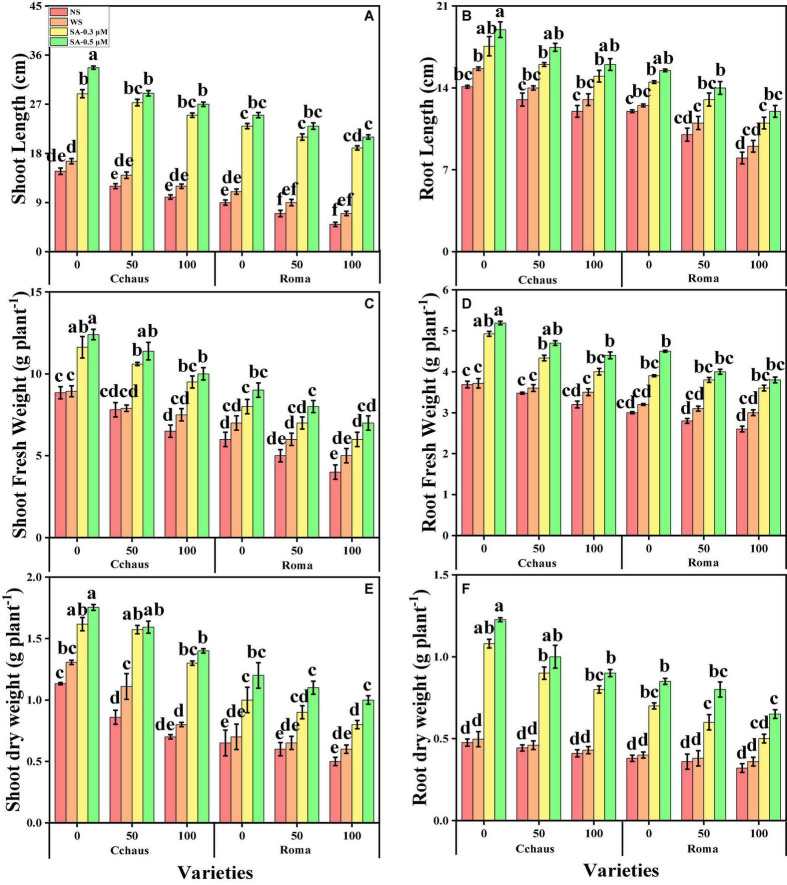
Effect of exogenously applied syringic acid (0.3-0.5°μM) on shoot length **(A)**, root length **(B)**, shoot fresh weight **(C)**, root fresh weight **(D)**, shoot dry weight **(E)**, and root dry weight **(F)** on *Solanum lycopersicum* varieties (Cchaus and Roma) cultivated under stress levels of Pb [0 (no Pb), 50 and 100 mg kg^–1^]. Values are demonstrated as means of four replicates along with standard deviation (SD; *n* = 4). Two-way ANOVA was performed and means differences were tested by HSD (*P* < 0.05) through Tukey’s test. Different lowercase letters on the error bars indicate significant difference between the treatments.

**FIGURE 2:**
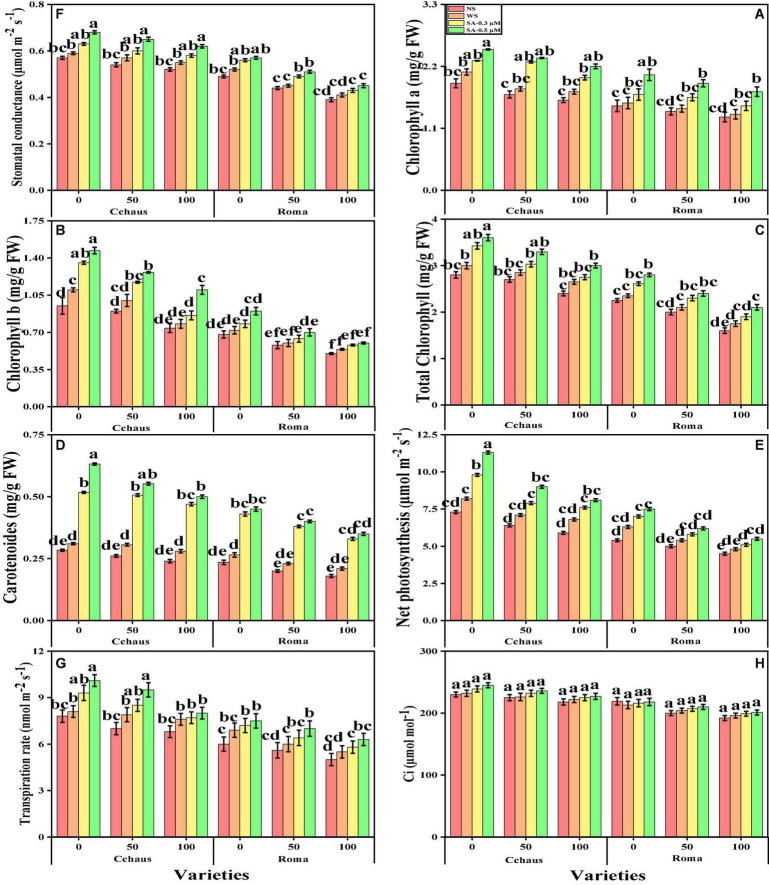
Effect of exogenously applied syringic acid (0.3-0.5°μM) on chlorophyll-a content **(A)**, chlorophyll-b contents **(B)**, total chlorophyll contents **(C)**, carotenoid contents **(D)**, net photosynthesis, **(E)** stomatal conductance **(F)**, transpiration rate **(G)**, and intercellular CO_2_ **(H)** on *Solanum lycopersicum* varieties (Cchaus and Roma) cultivated under stress levels of Pb [0 (no Pb), 50 and 100 mg kg^–1^]. Values are demonstrated as means of four replicates along with standard deviation (SD; *n* = 4). Two-way ANOVA was performed and means differences were tested by HSD (*P* < 0.05) through Tukey’s test. Different lowercase letters on the error bars indicate significant difference between the treatments.

In this study, we elucidated various growth parameters, photosynthetic pigments, and gas exchange characteristics under the various levels of syringic acid in Pb-polluted soil in *S. lycopersicum* varieties. We presented the various morphological traits of *S. lycopersicum* varieties in Figure 1 and photosynthetic pigments and gas exchange characteristics in Figure 2, which were grown in a Pb-polluted environment under the application of various levels of syringic acid. Our results depicted that all root length, shoot length, shoot fresh weight, root fresh weight, shoot dry weight, root dry weight, chlorophyll contents, carotenoid contents, net photosynthesis, stomatal conductance, and transpiration rate were decreased (non-significantly) with the increase in the Pb levels (50 and 100 mg kg^–1^) in the soil when compared with the plants grown without the addition of Pb in the soil in both *S. lycopersicum* varieties (Figures 1, 2). In addition, results from the present study also show that Cchaus showed more tolerance/resistance to the Pb stress in the soil compared to Roma at all levels of the Pb-stressed environment in the soil. We also noticed that various growth parameters, photosynthetic pigments, and gas exchange characteristics can be increased under the toxic concentration of Pb in the soil by the exogenous application of syringic acid (Figures 1, 2). In addition, results also show that exogenous application with syringic acid increased (significantly) all growth parameters, photosynthetic pigments, and gas exchange characteristics in both *S. lycopersicum* varieties, compared to those plants which were grown without the exogenous application with syringic acid in the soil.

### Effect of exogenous application of syringic acid on oxidative stress indicators in *Solanum lycopersicum* varieties under the toxic concentrations of Pb

**FIGURE 3:**
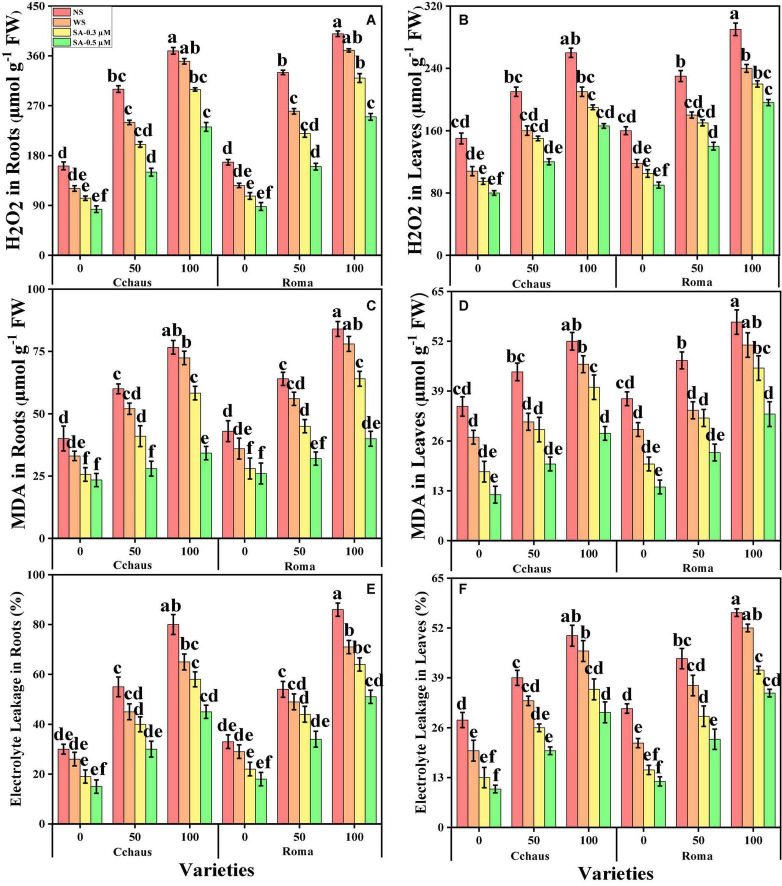
Effect of exogenously applied syringic acid (0.3-0.5°μM) on H_2_O_2_ contents in the roots **(A)**, H_2_O_2_ contents in the leaves **(B)**, malondialdehyde (MDA) contents in the roots **(C)**, MDA contents in the leaves **(D)**, electrolyte leakage (EL) percentage in the roots **(E)**, and EL percentage in the leaves **(F)** on *Solanum lycopersicum* varieties (Cchaus and Roma) cultivated under stress levels of Pb [0 (no Pb), 50 and 100 mg kg^–1^]. Values are demonstrated as means of four replicates along with standard deviation (SD; *n* = 4). Two-way ANOVA was performed and means differences were tested by HSD (*P* < 0.05) through Tukey’s test. Different lowercase letters on the error bars indicate significant difference between the treatments.

Oxidative stress markers i.e., malondialdehyde (MDA) contents, hydrogen peroxide (H_2_O_2_) initiation, and electrolyte leakage (%) in the roots and leaves of *S. lycopersicum* varieties grown in a toxic concentration of Pb in the soil were also measured in the present study. The results regarding MDA, H_2_O_2_, and EL in the roots and leaves of *S. lycopersicum* varieties grown under the application of syringic acid in Pb-polluted soil, are presented in Figure 3. From the given results, we also elucidated that increasing concentration of Pb in the soil induced a significant (*P* < 0.05) increase in the contents of MDA, H_2_O_2_ initiation and EL (%) in the roots and leaves of *S. lycopersicum* varieties, when compared with those plants which were grown without the addition of Pb concentration in the soil (Figure 3). Results also showed that the contents of MDA, H_2_O_2_ initiation, and EL (%) were significant (*P* < 0.05) higher in the roots, when compared to the shoots of *S. lycopersicum* varieties. In addition, Pb-sensitive genotype i.e., Roma showed higher values of MDA contents, H_2_O_2_ initiation, and EL (%) in all organs of the plants, in comparison with Pb-tolerant genotype i.e., Cchaus (Figure 3). Results also illustrated that the application of syringic acid decrease the contents of MDA, H_2_O_2_ initiation, and EL (%) in the roots and leaves of *S. lycopersicum* varieties, compared with those plants which were grown without the exogenous application with syringic acid. In addition, at all levels of Pb stress (50 and 100 mg Kg^–1^), the contents of MDA, H_2_O_2_ initiation and EL (%) decreased with the increasing levels of syringic acid (0.3-0.5°μM) in the soil, compared with those plants which were grown without the application of syringic acid.

### Effect of exogenous application of syringic acid on enzymatic and non-enzymatic antioxidants in *Solanum lycopersicum* varieties under the toxic concentrations of Pb

**FIGURE 4:**
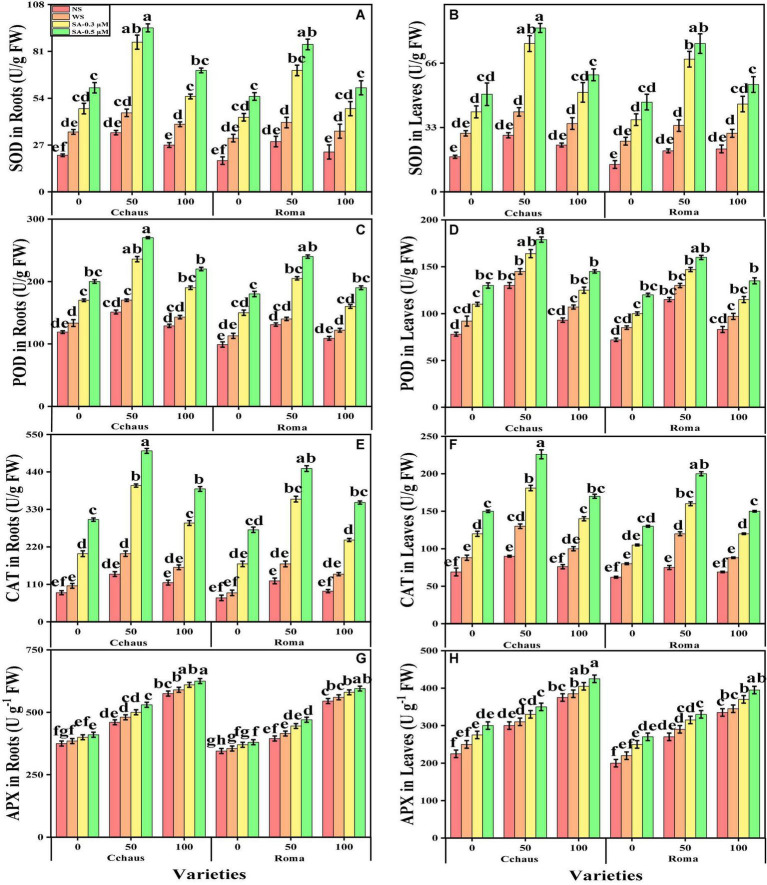
Effect of exogenously applied syringic acid (0.3-0.5°μM) on superoxide dismutase (SOD) activity in the roots **(A)**, SOD activity in the leaves **(B)**, peroxidase (POD) activity in the roots **(C)**, POD activity in the leaves **(D)**, CAT activity in the roots **(E)**, catalase (CAT) activity in the leaves **(F)**, ascorbate peroxidase (APX) activity in the roots **(G)** and APX activity in leaves **(H)** on *Solanum lycopersicum* varieties (Cchaus and Roma) cultivated under stress levels of Pb [0 (no Pb), 50 and 100 mg kg^–1^]. Values are demonstrated as means of four replicates along with standard deviation (SD; *n* = 4). Two-way ANOVA was performed and means differences were tested by HSD (*P* < 0.05) through Tukey’s test. Different lowercase letters on the error bars indicate significant difference between the treatments.

**FIGURE 5:**
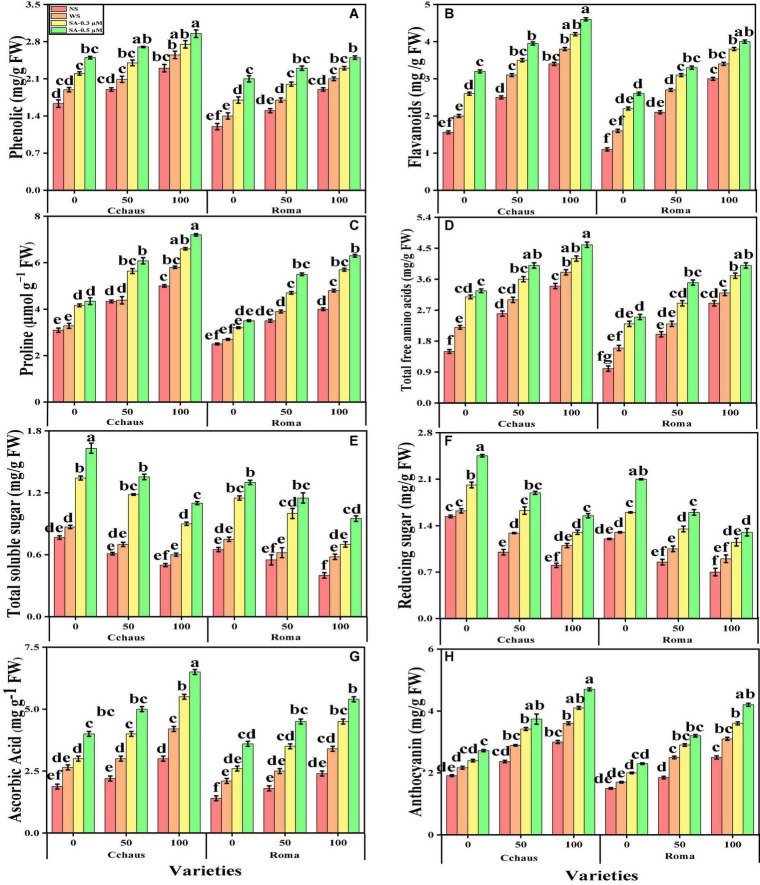
Effect of exogenously applied syringic acid (0.3-0.5°μM) on phenolic contents **(A)**, flavonoid contents **(B)**, proline contents **(C)**, total free amino acids **(D)**, soluble sugar contents **(E)**, reducing sugar contents **(F)**, ascorbic acid contents **(G)**, and anthocyanin contents **(H)** on *Solanum lycopersicum* varieties (Cchaus and Roma) cultivated under stress levels of Pb [0 (no Pb), 50 and 100 mg kg^–1^]. Values are demonstrated as means of four replicates along with standard deviation (SD; *n* = 4). Two-way ANOVA was performed and means differences were tested by HSD (*P* < 0.05) through Tukey’s test. Different lowercase letters on the error bars indicate significant difference between the treatments.

In the present study, we also measured various enzymatic antioxidants i.e., superoxidase dismutase (SOD), peroxidase (POD), catalase (CAT), and ascorbate peroxidase (APX) from the roots and leaves and non-enzymatic compounds i.e., phenolic contents, flavonoid contents, ascorbic acid contents, and also proline contents, total free amino acids, soluble sugar contents, reducing sugar contents and anthocyanin contents from the leaves of *S. lycopersicum* varieties grown under the application of syringic acid in Pb-polluted soil. The data regarding the activities of antioxidants (SOD, POD, CAT, and APX) in the roots and leaves and also non-enzymatic compounds, sugar, and proline of *S. lycopersicum* varieties grown under the application of syringic acid in Pb-polluted soil are presented in Figures 4, 5, respectively. According to the given results, we elucidated that increasing concentrations of Pb in the soil increased activities of antioxidants (SOD, POD, CAT, and APX) in the roots and leaves and also non-enzymatic compounds of *S. lycopersicum* varieties, compared with those plants which were grown without the addition of Pb in the soil. The activities of various antioxidants (SOD, POD, CAT, and APX) in the roots and leaves of *S. lycopersicum* varieties initially increased up to a Pb level of 50 mg Kg^–1^ in the soil, but further increased in Pb concentration in the soil (100 mg Kg^–1^) induced a significant (*P* < 0.05) decrease in antioxidants in the roots of leaves of both *S. lycopersicum* varieties (Figure 4). Results also showed that the activities of antioxidants (SOD, POD, CAT, and APX) were significantly (*P* < 0.05) higher in the roots, when compared to the shoots of *S. lycopersicum* varieties. In addition, Pb-tolerant genotypes i.e., Cchaus, showed higher activities of antioxidants (SOD, POD, CAT, and APX) in the root and leaves of the plants, when compared with Pb-sensitive genotypes i.e., Roma (Figure 4). Results also showed that the sugar contents were decreased by increasing the concentration of Pb in the soil while the content of proline in the leaves was increased by increasing the concentration of Pb in the soil in both varieties of *S. lycopersicum* (Figure 5). Results also illustrated that the application of syringic acid increased the activities of enzymatic antixoidants (SOD, POD, CAT, and APX) in the roots and leaves of both varieties of *S. lycopersicum* (Roma and Cchaus), compared with those plants which were grown without the exogenous application with syringic acid (Figure 4). In addition, at all levels of Pb stress (50 and 100 mg Kg^–1^), the activities of antioxidants (SOD, POD, CAT, and APX) were increased with the increasing levels of syringic acid in the soil, compared with those plants which were grown without the application of syringic acid (Figure 4).

### Effect of exogenous application of syringic acid on nutrient uptake, organic acid, and Pb uptake in *Solanum lycopersicum* varieties under the toxic concentrations of Pb

**FIGURE 6:**
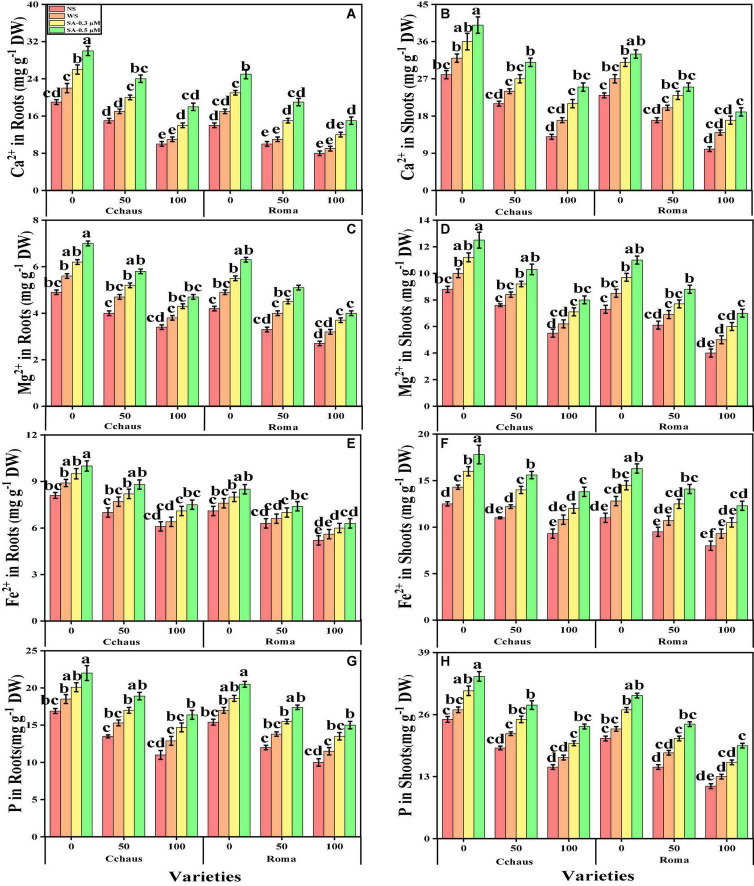
Effect of exogenously applied syringic acid (0.3-0.5°μM) on calcium contents in the roots **(A)**, calcium contents in the shoots **(B)** magnesium contents in the roots **(C)**, magnesium contents in the shoots **(D)**, iron contents in the roots **(E)**, iron contents in the shoots **(F)**, phosphorus contents in the roots **(G)**, and phosphorus contents in the shoots **(H)** on *Solanum lycopersicum* varieties (Cchaus and Roma) cultivated under stress levels of Pb [0 (no Pb), 50 and 100 mg kg^–1^]. Values are demonstrated as means of four replicates along with standard deviation (SD; *n* = 4). Two-way ANOVA was performed and means differences were tested by HSD (*P* < 0.05) through Tukey’s test. Different lowercase letters on the error bars indicate significant difference between the treatments.

In the present study, the contents of essential minerals i.e., iron (Fe^2+^), magnesium (Mg^2+^), calcium (Ca^2+^), and phosphorus (P) were also determined from the roots and shoots of *S. lycopersicum* varieties grown in different application levels of syringic acid under Pb-polluted soil. The contents of Fe^2+^, Ca^2+^, Mg^2+^, and P from the roots and shoots of *S. lycopersicum* varieties are presented in Figure 6. Our results depicted that the concentrations of Fe^2+^, Ca^2+^, Mg^2+^ and P in the roots and shoots of *S. lycopersicum* varieties were decreased with the increase in the Pb levels (50 and 100 mg kg^–1^) in the soil when compared with the plants grown without the addition of Pb in the soil in both *S. lycopersicum* varieties (Figure 6). In addition, results from the present study also show that Cchaus showed more concentrations of Fe^2+^, Ca^2+^, Mg^2+^, and P in the roots and shoots of the plants compared to Roma at all levels of the Pb-stressed environment in the soil. We also noticed that the concentrations of Fe^2+^, Ca^2+^, Mg^2+,^ and P in the roots and shoots of *S. lycopersicum* varieties can be increased under the toxic concentration of Pb in the soil by the exogenous application of syringic acid (Figure 6). In addition, results also show that exogenous application with syringic acid increased the concentrations of Fe^2+^, Ca^2+^, Mg^2+^, and P in the roots and shoots of *S. lycopersicum* varieties, compared to those plants which were grown without the exogenous application with syringic acid in the soil.

**FIGURE 7:**
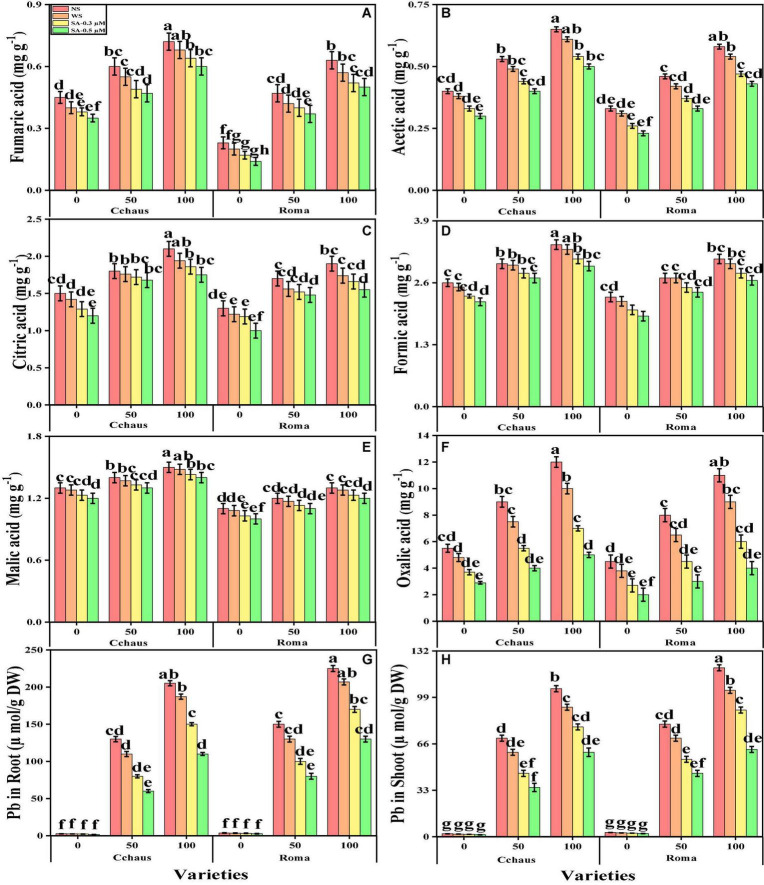
Effect of exogenously applied syringic acid (0.3-0.5°μM) on fumaric acid contents **(A)**, acetic acid contents **(B)**, citric acid contents **(C)**, formic acid contents **(D)**, malic acid contents **(E)**, oxalic acid contents **(F)**, Pb concentration in the roots **(G)** and Pb concentration in the shoots **(H)** on *Solanum lycopersicum* varieties (Cchaus and Roma) cultivated under stress levels of Pb [0 (no Pb), 50 and 100 mg kg^–1^]. Values are demonstrated as means of four replicates along with standard deviation (SD; *n* = 4). Two-way ANOVA was performed and means differences were tested by HSD (*P* < 0.05) through Tukey’s test. Different lowercase letters on the error bars indicate significant difference between the treatments.

The contents of fumaric acid, formic acid, acetic acid, citric acid, malic acid, and oxalic acid in the roots of both *S. lycopersicum* varieties grown under toxic levels of Pb in the soil, with or without the application of syringic acid are presented in Figure 7. According to the given results, we have noticed that increasing the concentration of Pb in the nutrient solution (50 and 100 mg kg^–1^) induced a significant (*P* < 0.05) increase in the contents of fumaric acid, formic acid, acetic acid, citric acid, malic acid, and oxalic acid in the roots of both *S. lycopersicum* varieties, compared to those plants which were grown in Pb level of 0 mg kg^–1^ in the soil. Results also illustrated that the application of syringic acid decrease the contents of fumaric acid, formic acid, acetic acid, citric acid, malic acid, and oxalic acid in the roots of *S. lycopersicum* varieties, compared with those plants which were grown without the exogenous application with syringic acid. In addition, at all levels of Pb stress (50 and 100 mg kg^–1^), the contents of fumaric acid, formic acid, acetic acid, citric acid, malic acid, and oxalic acid were decreased with the increasing foliar levels of syringic acid, compared with those plants which were grown without the application of syringic acid.

We also manifested the contents of Pb from the roots and shoots of *S. lycopersicum* varieties grown under toxic levels of Pb in the soil, with or without the application of syringic acid are presented in Figure 7. Increasing levels of Pb in the soil, induce a significant (*P* < 0.05) increase in the Pb concentration in the roots and shoots of *S. lycopersicum* varieties, compared to those plants which were grown in the control treatment. In addition, the Pb-sensitive genotype i.e., Roma showed higher contents of Pb in the roots and shoots of the plants, in comparison with the Pb-tolerant genotype i.e., Cchaus (Figure 7). In addition, at all levels of Pb stress (50 and 100 mg kg^–1^), the contents of Pb were decrease with the increasing levels of syringic acid in the nutrient solution, compared with those plants which were grown without the application of syringic acid.

### Correlation between morpho-physiological traits of *Solanum lycopersicum* and Pb accumulation

**FIGURE 8:**
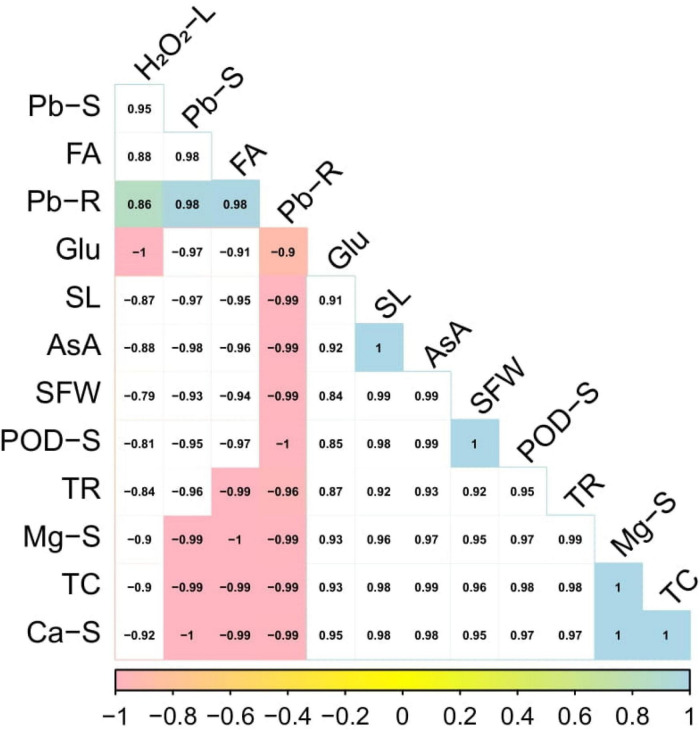
Relationship of various growth attributes of *Solanum lycopersicum* (Cchaus) with Pb uptake under various application levels of syringic acid grown in Pb-contaminated soil. Different abbreviations used in the figure are as follows: Pb-S, lead concentration in the shoots; FA, fumaric acid; Pb-R, lead concentration in the roots; Glu, glucose content; SL, shoot length; AsA, ascorbic acid content; SFW, shoot fresh weight; POD-S, peroxidase activity in the shoots; TR, transpiration rate; Mg-S, magnesium content in the shoots; TC, total chlorophyll content; and Ca-S, calcium content in the shoots.

A Pearson’s correlation histogram was also illustrated to depict a correlation between Pb uptake with growth and composition by *S. lycopersicum* under various application levels of syringic acid grown in the Pb-contaminated soil (Figure 8). Although, both varieties showed the same trend, so we constructed only one graph (histogram-correlation analysis) of Cchaus. Pb concentration in the roots was positively correlated with Pb concentration in the shoots, fumaric acid, peroxidase activity in the shoots, hydrogen peroxide in the leaves while negatively correlated with glucose content, shoot length, ascorbic acid content, shoot fresh weight, transpiration rate, magnesium content in the shoots, total chlorophyll content, and calcium content in the shoots. Similarly, Pb concentration in the shoots was positively correlated with Pb concentration in the roots, fumaric acid, peroxidase activity in the shoots, and hydeogen peroxide in the leaves while negatively correlated with glucose content, shoot length, ascorbic acid content, shoot fresh weight, transpiration rate, magnesium content in the shoots, total chlorophyll content, and calcium content in the shoots. This relationship showed a close connection between plant growth of *S. lycopersicum* grown in various application levels of syringic acid under Pb-toxic soil.

### Principal component analysis

**FIGURE 9:**
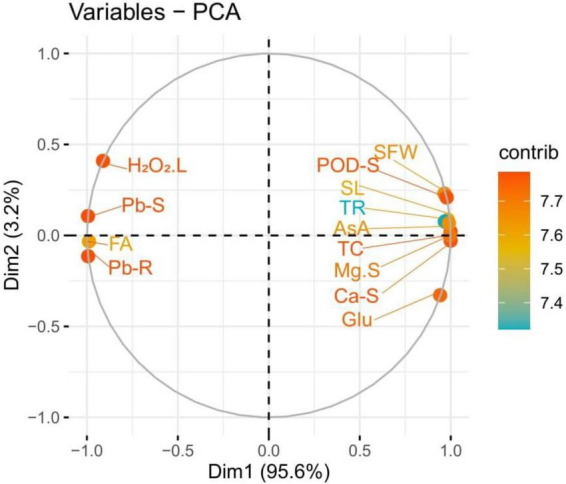
Loading plots of principal component analysis (PCA) of various growth attributes of *Solanum lycopersicum* with Pb uptake under various application levels of syringic acid grown in Pb-contaminated soil. Different abbreviations used in the figure are as follows: Pb-S, lead concentration in the shoots; FA, fumaric acid; Pb-R, lead concentration in the roots; Glu, glucose content; SL, shoot length; AsA, ascorbic acid content; SFW, shoot fresh weight; POD-S, peroxidase activity in the shoots; TR, transpiration rate; Mg-S, magnesium content in the shoots; TC, total chlorophyll content; and Ca-S, calcium content in the shoots.

The loading plots of principal component analysis (PCA) to illustrate the effect of syringic acid in the various levels of Pb toxicity in *S. lycopersicum* are presented in Figure 9. Although, both varieties show the same trend, so we constructed only one graph (PCA) of Cchaus. In the whole database, Dim1 and Dim2 exhibited the maximum portion and occupied more than 98.8% in all databases. Among which Dim1 exhibited (95.6.8%) and Dim2 exhibited (3.2%). All studied parameters were distributed successfully in the database which is giving a clear indication that Pb stress causes a significant effect on the eco-physiology of *S. lycopersicum*. From the results, it can be indicated that Pb concentration in the roots, Pb concentration in the shoots, fumaric acid, and hydrogen peroxide were negatively correlated in the database to all other parameters studied in this experiment. While, glucose content, shoot length, ascorbic acid content, shoot fresh weight, peroxidase activity in the shoots, transpiration rate, magnesium content in the shoots, total chlorophyll content, and calcium content in the shoots were positively correlated with all other studied attributes.

## Discussion

Heavy metal contamination of agricultural lands has become an emerging environmental issue worldwide (Mallhi et al., 2019; Zaheer et al., 2020). This problem is becoming a serious threat to humans and animals due to the entry of heavy metals into the food web. High concentrations of heavy metals significantly reduce plant productivity and crop yields (Saleem et al., 2020; Alsafran et al., 2021; Al Jabri et al., 2022). Pb is an extremely phytotoxic heavy metal that induces several physiological and ultrastructural disorders in plants (Niazy Abdou and Wahdan, 2017). Generally, sensitivity to metal toxicity in plants relies on metal concentration, exposure time, plant species, age, and tissue type (Ali et al., 2014). The decrease in plant biomass (Figure 1) likely indicates heavy metal stress caused by the interference of Pb with the plant physiology and metabolism (Wen et al., 2020; Hussain et al., 2022). In particular, a reduction in protein synthesis (Zulfiqar et al., 2019) and photosynthesis (Figure 2), and damage to the cell and sub-cellular organelles (Kanwal et al., 2014). We also manifested that, under the same stress levels of Pb (50 and 100 mg kg^–1^), Cchaus was more tolerant to Pb stress than Roma. The differences in growth in different varieties of *S. lycopersicum* under the same stress condition might be due to the low availability of water contents, poor stomatal conductance, and alterations in root architecture (Afzal et al., 2020; Saleem et al., 2020, 2020d). Additionally, Pb stress induced a negative impact on leaf chlorophyll contents and gas exchange attributes. This might be the consequence of disruption of chloroplast, protein complex, and photosynthetic apparatus when plants are exposed to heavy metal stress (Shafigh et al., 2016; Uddin et al., 2016).

Heavy metals are considered a primary source of injury to the cell membrane, frequently attributed to lipid peroxidation. Excessive reactive oxygen species (ROS) production causes oxidative stress, as reported for many crops under heavy metals treatment, and is likely to be commenced by molecular oxygen excitation (O_2_) to generate singlet oxygen or by electron transfer to O_2_ and genesis of free radicals, i.e., O^2–^ and OH^–^ (Bharwana et al., 2013; Amooaghaie and Enteshari, 2017). Plant response to oxidative stress also depends upon plant species and cultivars, and this ROS production in plants is removed by a variety of antioxidant enzymes such as SOD, POD, CAT, and APX (Imran et al., 2021; Alam et al., 2022). Plants produce a variety of secondary metabolites such as proline, flavonoids, and phenolics that improve tolerance against metal toxicity (Imran et al., 2019). In the present experiment, the reduction of enzyme activity (Figure 4) under the severe Pb levels toxicity is due to a delay in the elimination of H_2_O_2_ and toxic peroxides intervened by POD and APX (Qin et al., 2018). In addition, the decrease in antioxidants at extremely high Pb concentration in the soil (100 mg kg^–1^) might be due to alterations in gene expression and functions of some proteins in different plant organs (Ali et al., 2014; Kanwal et al., 2014).

Essential nutrients are required for the normal growth of plants. Numerous reports demonstrated that the uptake and translocation of essential elements in plants were restricted under heavy metal stress (Hashmat et al., 2021; Zaheer et al., 2022). Excess Pb decreased the Ca^2+^, Mg^2+^, Fe^2+^, and P contents in numerous plant species, which may cause ions deficiency in plants. In the present research, it was observed that Pb toxicity reduced the Ca^2+^, Mg^2+^, Fe^2+^, and P contents in shoots and roots of *S. lycopersicum* as compared to those plants grown in soil without the addition of Pb (Figure 7). Pb-toxicity in plants depends upon the quantity of Pb available for plants and intensity of those elements which have the capability to compete with Pb (Shahid et al., 2014; Adnan et al., 2022; Ahmad et al., 2022a). Reduced Ca^2+^, Mg^2+^, Fe^2+^, and P contents in plants are due to its inability to uptake essential nutrients in the presence of elevated levels of Pb in soil (Zulfiqar et al., 2019). Roots exclude especially organic acids, which are regarded as active ligands under the excess concentration of metals in the soil (Javed et al., 2017). Acidification of mucilage after uptake of Pb is likely due to the release of protons when plant roots release more cations than anions in order to maintain their charge balance (Figure 7).

**FIGURE 10:**
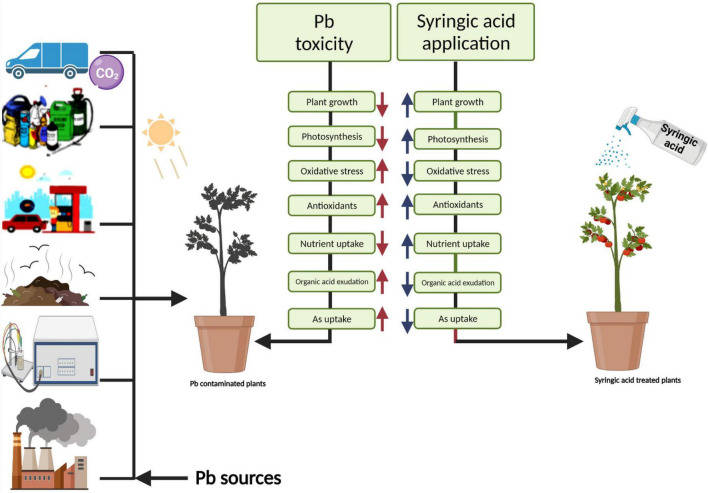
Schematic presentation interpreting the mechanistic role of syringic acid in the alleviation of lead toxicity in *Solanum lycopersicum*. Pb toxicity inhibits plant growth characteristics and higher oxidative stress, organic acid exudation, and Pb concentration in the roots and shoots of *S. lycopersicum*. In contrast, the application of syringic acid alleviated Pb toxicity and improved plant growth and biomass, photosynthetic efficiency, antioxidant defense system, and nutritional status of the plant. In addition, the application of syringic acid decreases the contents of organic acid, oxidative damage in the plant tissues, and Pb contents in the roots and shoots of *S. lycopersicum*. The current study demonstrated that syringic acid could relieve Pb toxicity in *S. lycopersicum* by reducing Pb concentration in the various parts of plants and regulating the nutritional status of the plant.

Different practices have been used for the management of Pb-contaminated soils and their reduction in crop plants. The use of phytochemicals such as syringic acid in the control of human diseases and under abiotic stresses in the plants has seem considerable public and scientific interest of late (Srinivasulu et al., 2018). Syringic acids are methoxylated aromatic compounds that often serve as models of the subunits of lignin. Although syringic acid has important implications for global carbon cycles, there is limited information on its fate in anoxic environments (Phelps and Young, 1997). Employment of syringic acid for the remediation of Pb-toxicity is a novel technique, which is a phenolic compound and occurs abundantly in plants and some fungi (Srinivasulu et al., 2018). The application of syringic acid to plants can enhance levels of antioxidants, which contribute significantly to the alleviation of oxidative stress (Vo et al., 2020). It protects plants from injurious impacts of oxidative stress such as protein and lipid oxidation (Srinivasulu et al., 2018). Recent studies reported that syringic acid can alleviate stunted growth caused by cesium stress in *Arabidopsis* (Adams et al., 2020). In this research work, treatment of *S. lycopersicum* with syringic acid enhanced growth parameters (Figure 1) and improved the activities of photosynthetic apparatus (Figure 2) by regulating and enhancing the uptake of essential nutrients (Figure 6) through plants reducing oxidative stress (Figure 3) by managing the activities of enzymatic and non-enzymatic antioxidants (Figures 4, 5). This is because syringic acid regulates the uptake of sufficient essential nutrients and minimizes oxidative stress in plants (Vo et al., 2020). There is very limited literature, which represents the utilization of syringic acid for the remediation of side effects caused by heavy metals. Results of present research indicate that it will be a valuable approach to utilize syringic acid to cope with heavy metal stress in agricultural crops. The complete description of Pb toxicity and its alleviation using the application of syringic acid in *S. lycopersicum* is presented in Figure 10.

## Conclusion

Outcomes of the current study revealed that toxic levels of Pb significantly affected plant growth and biomass, photosynthetic pigments, gaseous exchange traits, antioxidative machinery, and minerals uptake by *S. lycopersicum* genotypes. Furthermore, Pb toxicity increased the oxidative stress indicators, organic acid exudations, and Pb contents in plant organs. We also noticed that under the same stress levels of Pb, Cchaus was more tolerant to Pb stress than Roma. However, Si syringic acid improved plant growth and biomass, decreased ROS production, maintain essential minerals, and decreased the Pb contents of plant organs. Furthermore, balanced exudation of organic acids after syringic acid supplementation further confers the normal metabolic activities of *S. lycopersicum* genotypes even under Pb stress. Therefore, long-term field studies should be executed to draw parallels amongst plants/crops’ root exudations, metal stress, nutrient mobility patterns, and plant growth in order to gain insights into underlying mechanisms.

## Data availability statement

The raw data supporting the conclusions of this article will be made available by the authors, without undue reservation.

## Author contributions

IH, SE, and MA: conceptualization. IH, RR, MA, and SM: data curation. IH, BA, DV, RR, HA, MA, and SM: formal analysis. SE, RM, and DV: funding acquisition. RR, BA, and SM: investigation. IH and MA: methodology. HA, ME, SA, AA, SM, DV, and RM: project administration. RM and DV: resources. SA, AA, ME, MS, BA, JM, and DV: software. IH and SM: supervision. HA, SE, JM, RM, MS, and SM: writing – original draft. MS, HA, BA, SA, AA, JM, ME, and RM: writing – review and editing. All authors contributed to the article and approved the submitted version.
